# Assessment of Subcellular ROS and NO Metabolism in Higher Plants: Multifunctional Signaling Molecules

**DOI:** 10.3390/antiox8120641

**Published:** 2019-12-12

**Authors:** Sukhmeen Kaur Kohli, Kanika Khanna, Renu Bhardwaj, Elsayed Fathi Abd_Allah, Parvaiz Ahmad, Francisco J. Corpas

**Affiliations:** 1 Department of Botanical and Environmental Sciences, Guru Nanak Dev University, Amritsar, Punjab 143005, India; sukhmeenkohli@gmail.com (S.K.K.); kanika.27590@gmail.com (K.K.); 2 Department of Plant Production, College of Food & Agricultural Sciences, King Saud University, Riyadh 11451, Saudi Arabia; 3 Botany and Microbiology Department, College of Science, King Saud University, Riyadh 11451, Saudi Arabia; 4 Department of Botany, S.P. College, Srinagar 190001, India; 5 Department of Biochemistry, Cell and Molecular Biology, Estación Experimental del Zaidín, Consejo Superior de Investigaciones Científicas (CSIC), C/Profesor Albareda, 118008 Granada, Spain

**Keywords:** antioxidants, reactive oxygen species, nitric oxide, organelles, signaling, stress

## Abstract

Reactive oxygen species (ROS) and nitric oxide (NO) are produced in all aerobic life forms under both physiological and adverse conditions. Unregulated ROS/NO generation causes nitro-oxidative damage, which has a detrimental impact on the function of essential macromolecules. ROS/NO production is also involved in signaling processes as secondary messengers in plant cells under physiological conditions. ROS/NO generation takes place in different subcellular compartments including chloroplasts, mitochondria, peroxisomes, vacuoles, and a diverse range of plant membranes. This compartmentalization has been identified as an additional cellular strategy for regulating these molecules. This assessment of subcellular ROS/NO metabolisms includes the following processes: ROS/NO generation in different plant cell sites; ROS interactions with other signaling molecules, such as mitogen-activated protein kinases (MAPKs), phosphatase, calcium (Ca^2+^), and activator proteins; redox-sensitive genes regulated by the iron-responsive element/iron regulatory protein (IRE-IRP) system and iron regulatory transporter 1(IRT1); and ROS/NO crosstalk during signal transduction. All these processes highlight the complex relationship between ROS and NO metabolism which needs to be evaluated from a broad perspective.

## 1. Introduction

Molecular oxygen (O_2_), which is essential for life, is formed naturally by various aerobic metabolic processes. Reactive oxygen species (ROS) are redox molecules mainly produced in aerobic organisms, with some of the most important being photosynthetic cells, in which ROS production needs to be continually regulated to prevent undesirable functional collateral damage. ROS, which are involved in signaling processes in subcellular compartments and cells, as well as in mechanisms of defense against biotic and abiotic stresses, are also essential for cell survival [1,2].

**Figure 1:**
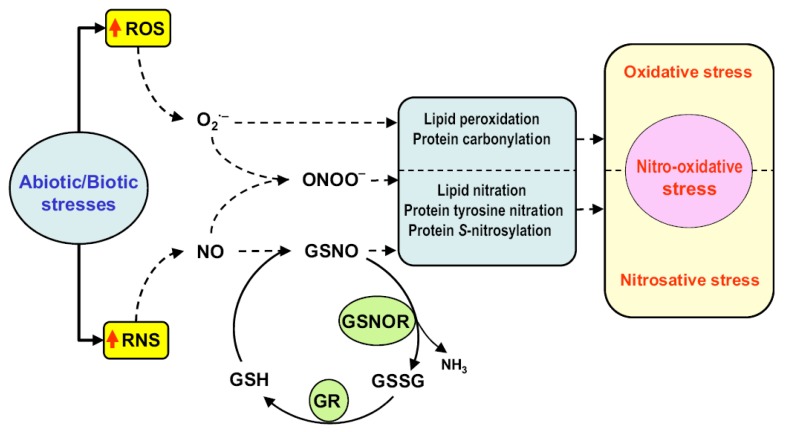
Simple model of the interplay between reactive oxygen species (ROS) and reactive nitrogen species (RNS) in response to biotic and abiotic stresses in plant cells which could have associated a nitro-oxidative stress. Reproduced with permission from Corpas and Barroso (2013) New Phytologist 11: 535-542. John Wiley and Sons and Copyright Clearance Center. Abbreviations: GR—glutathione reductase. GSNO—*S*-nitrosoglutathione. GSH—glutathione. GSSG—glutathione disulfide. O_2_^•−^—superoxide anion. NH_3_—ammonia. NO—nitric oxide. ONOO^−^—peroxynitrite. ROS—reactive oxygen species. RNS—reactive nitrogen species.

In higher plants, the principal subcellular ROS production sites include chloroplasts, mitochondria, cytosols, vacuoles, peroxisomes, plasma membranes, apoplasts, and cell walls [3]. ROS, such as hydrogen peroxide (H_2_O_2_), superoxide anion (O_2_^•−^), hydroxyl radical (^•^OH), and singlet oxygen (^1^O_2_), are metabolically closely associated with another family of molecules, called reactive nitrogen species (RNS), including peroxynitrite (ONOO^−^) and *S*-nitrosoglutathione (GSNO), derived from the nitric oxide (^•^NO) radical. Though generated under normal physiological conditions, these two molecular families are prone to uncontrolled overproduction under stressful circumstances, causing cellular nitro-oxidative damage which can hamper cell survival [4,5]. Figure 1 shows a simple model of ROS/RNS interplay in response to biotic and abiotic stresses, including nitro-oxidative stress, in plant cells. Usually associated with cytotoxicity, ROS/RNS also act as signal molecules involved in cellular interactions and mechanisms [6,7]. 

ROS interact with many other signaling molecules which influence a broad spectrum of responses. More advanced transcriptomic techniques have identified important stress-responsive signaling molecules, such as mitogen-activated protein kinase (MAPK)and phosphatase, involved in ROS metabolism [8]. Plant MAPK signaling networks, which differentiate between stresses through receptors and sensors present on the cell surface, are subjected to transduction [9]. Calcium (Ca^2+^) is also actively involved in intra- and extra-cellular signaling networks [10]. Ca^2+^ and ROS signaling interactions are considered to be bidirectional, as Ca^2+^ is necessary for ROS production, while ROS is primarily required for the regulation of cellular Ca^2+^ [11,12]. Stressed plant cells transduce signals via redox sensitive proteins and reversible redox reactions [13]. The iron-responsive element-iron-regulatory protein (IRE-IRP) system and iron-regulated transporter 1 (IRT1) are involved in the acclimation responses of stressed plant cells [14]. Furthermore, NO is considered to be a simple signal molecule with targets including thiols and the iron and sulfur group containing compounds and proteins [15]. The presence of NO in higher plants is well documented. However, how NO is enzymatically generated remains a matter of debate [16,17,18]. Up to now, two possible pathways have been recognized using as substrate either L-arginine (oxidative pathway) or nitrate/nitrite (reductive pathway) involving the contribution of a l-Arg-dependent NO synthase-like activity [19] and a nitrate reductase (NR) activity [20], respectively.

## 2. Overview of Principal Subcellular Compartments Involved in ROS/NO Production

**Figure 2:**
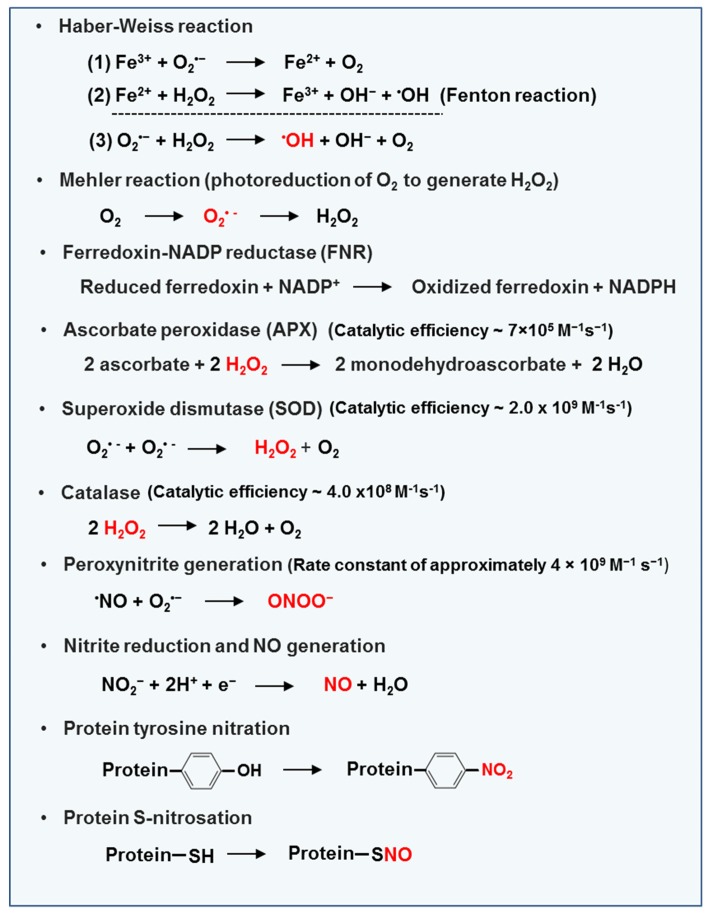
Key reactions of ROS and NO metabolism. Abbreviations: Fe^3+^—ferric ion. O_2_^•−^—superoxide anion. Fe^2+^—ferrous ion. H_2_O_2_—hydrogen peroxide. OH^−^—hydroxide. ^•^OH—hydroxyl ion. O_2_—molecular oxygen. NADP/ NADP^+^—nicotinamide adenine dinucleotide phosphate. FNR—ferredoxin-NADP reductase. NADPH—nicotinamide adenine dinucleotide phosphate. APX—ascorbate peroxidase. H_2_O—water. SOD—superoxide dismutase. ^•^NO—nitric oxide. ONOO^−^—peroxynitrite. NO_2_^−^—nitrite. H^+^—hydrogen ion.

Living organisms survive in an oxidizing environment where aerobic metabolism is supported by O_2_. ROS are produced from oxygen under physiological conditions in response to abiotic and biotic factors, which eventuallyreducesO_2_ to water containing physiologically important inter medium molecules such as H_2_O_2_, O_2_^•−^and ^•^OH [21]. Attenuated or transient alterations in the cellular redox state occur in response to ROS accumulation in stressed cells. Oxidative stress is initiated by the generation of O_2_^•−^, which is dismutated either chemically or enzymatically to H_2_O_2_ and then converted to ^•^OH via a Haber-Weiss reaction triggered by iron ions (Fe^2+^/Fe^3+^) [22] (Figure 2). Despite containing an O_2_^•−^pool of less than 0.5%, the protonated superoxide radical HO_2_^•^, also called a hydroperoxyl or perhydroxyl radical, is comparatively more toxic [23]. ^1^O_2,_ another toxic ROS species, is an electronically excited O_2_ generated in chloroplasts [21], which is regulated by an antioxidant located close to each ROS production site. This system has two main functions: to avoid potential oxidative damage and to perform signaling processes, especially in the case of H_2_O_2_ and NO. For example, H_2_O_2_ acts as a retrograde signal from chloroplasts and mitochondria to the nucleus and triggers an appropriated response [6]. Although more is known about ROS in higher plants, our knowledge of RNS, which perform a similar function, is growing. The principal sites and pathways involved in ROS/RNS production in plant cells, as well as key reactions, are summarized in Table 1 and Figure 2**,** respectively.

Despite excellent reviews focusing on plant ROS and NO metabolism, this assessment aims to provide mainly researchers without specialist knowledge in the field with an up-to-date, multifaceted, and comprehensive overall view of the literature.

### 2.1. Chloroplasts

Chloroplasts, the most common organelles found in plants, are equipped with all the biochemical machinery necessary to transform light energy into chemical energy through photosynthesis, one of the primary sources of ROS generation.

#### 2.1.1. ROS (O_2_^•−^, H_2_O_2_, and ^1^O_2_) and NO production sites

**Figure 3:**
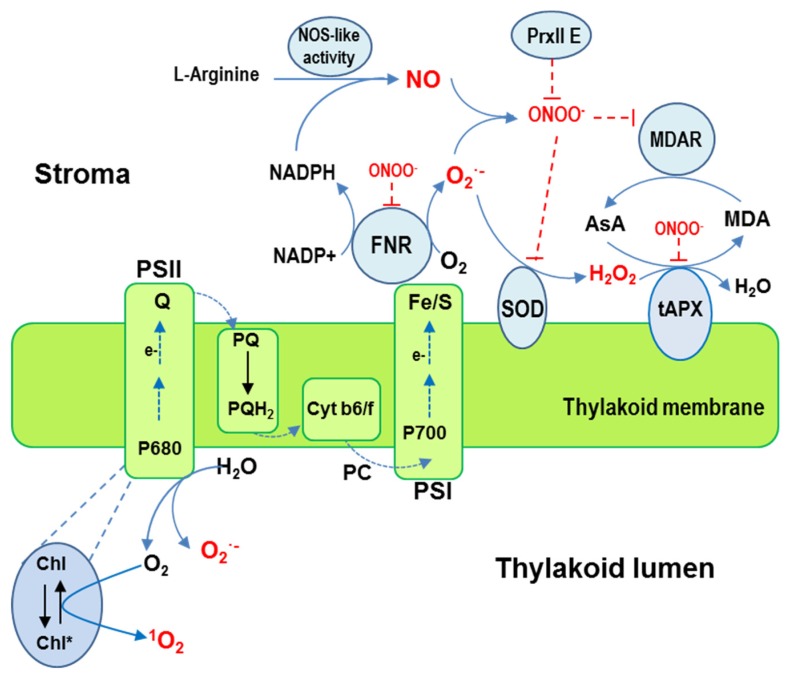
Simple model of ROS and NO generation in chloroplasts. O_2_^•^^−^is produced by the autoxidation of reduced ferredoxin at the photosystem I (PSI) and the plastoquinone (PQ) level in photosystem PSII, and then dismutated into H_2_O_2_ by superoxide dismutase (SOD). Abbreviation: Chl/ Chl*—chlorophyll. Cyt b6/7—Cytochrome b6f contains seven prosthetic groups. e^−^—electron. Fe/S—Iron/Sulfur. FNR—ferredoxin-NADP reductase. H_2_O_2_—hydrogen peroxide.MDA—monodehydroascorbate. O_2_—molecular oxygen. ^1^O_2_—singlet oxygen. H_2_O—water. O_2_^•^—superoxide anion. ONOO^−^—peroxynitrite. PC—plastocyanin. PS I—photosystem I. PSII—photosystem II. p680/p700—photosystem II primary donors. PQ—plastoquinone. PQH_2_—plastoquinol.Q—quinol. NADPH/NADP+—nicotinamide adenine dinucleotide phosphate. NO—nitric oxide. PRX II—peroxiredoxin II. AsA—ascorbate. MDAR—monodehyroascorbate reductase. SOD—superoxide dismutase. tAPX—thylakoid-bound ascorbate peroxidases.

ROS production in chloroplasts is directly related to photosynthetic light reactions in the thylakoid membrane. O_2_**^•−^** is fundamentally produced by autooxidation of reduced ferredoxin at photosystem I (PSI) and plastoquinone (PQ) level in photosystem PSII and then dismutated to H_2_O_2_ (Figure 3).

Under physiological conditions, the electron transport chain (ETC) from excited photosystem centers of the thylakoidal membrane is directed to NADP^+^ to enable ferredoxin-NADP reductase (FNR) to produce NADPH at PSI. Under stress conditions, the electron transfer chain (ETC) is overloaded, with part of the electron directed to O_2,_ causing O_2_^•−^ to be generated at PSI (Mehler reaction). O_2_^•−^ is dismutated to H_2_O_2_ either spontaneously or by superoxide dismutase (SOD) in the thylakoid membrane. It is worth pointing out that the highly efficient SOD-catalyzed dismutation reaction occurs at a diffusion-limited rate of ∼2 × 10^9^ M^−1^s^−1^, or ∼10^4^ times the rate constant for spontaneous dismutation, and H_2_O_2_ is then rapidly detoxified by ascorbate peroxidase (APX). The electron flow from H_2_O at PSII to H_2_O at PSI is called the water-water cycle [24]. This efficient cycle mechanism shortens the lifetime of photoproduced O_2_^•−^ and H_2_O_2_ to suppress the production of ^•^OH radicals, whose interaction with target molecules is thus prevented, leading to photoinhibition. This outcome is particularly important under stress conditions, such as high temperatures, drought and salinity. Depending on the plant species, several chloroplastic SOD isozymes, including Fe-SODs and Cu/Zn-SODs, bind to the thylakoid membrane located close to PSI [24,25]. Similarly, various APX isozymes are located either in the stroma (sAPX) or bind to the thylakoid membrane (tAPX) [26]. 

^1^O_2_, whose formation is unique to the chloroplast, is generated in the thylakoid membrane, specifically through the transfer of energy from P680, the primary electron donor at PSII, to ground O_2_ [24]. 

NO, which has been detected in chloroplasts, is generated by L-arginine- and NADPH-dependent NO synthase activity [27]. It stimulates chlorophyll biosynthesis and chloroplast differentiation [28,29] and mediates the PTMs, tyrosine nitration, and *S*-nitrosation, which affect chloroplast function [30]. The nitrated proteins identified in chloroplasts include FNR, β-carbonic anhydrase, glyceraldehyde-3-phosphate dehydrogenase, PSII light-harvesting complex II, methionine synthase, and Fe-SOD3 (FSD3), as well as subunits PsbO and PsbP of PSII lumen-exposed extrinsic proteins [31]. The *S*-nitrosated proteins identified include Rubisco, glyceraldehyde-3-phosphate dehydrogenase, dehydroascorbate reductase (DHAR), peroxiredoxin II E, fructose-1,6-bisphosphatase, phosphoribulokinase, and FNR [16,32,33]. All these data indicate that NO performs a regulatory function with respect to the chloroplast.

#### 2.1.2. Enzymatic and Non-Enzymatic Antioxidants

As mentioned above, O_2_^•−^ overproduction, which is regulated by stromal and thylakoid-bound SODs, is dismutated to H_2_O_2_ and then regulated by the APX cycle as part of the water-water cycle. When H_2_O_2_ content, estimated at roughly 0.5µM under normal conditions, is exposed to differential stress, it increases to 1–15 µM. Thus, regulation of H_2_O_2_, which triggers retrograde signaling from chloroplasts to the nucleus, especially under stress conditions, is of great importance in preventing oxidative damage [34]. The chloroplast contains other antioxidant systems to regulate H_2_O_2_, mainly the ascorbate-glutathione (ASC-GSH) cycle, peroxiredoxins (Prxs), and thioredoxins (Trxs).

**Figure 4:**
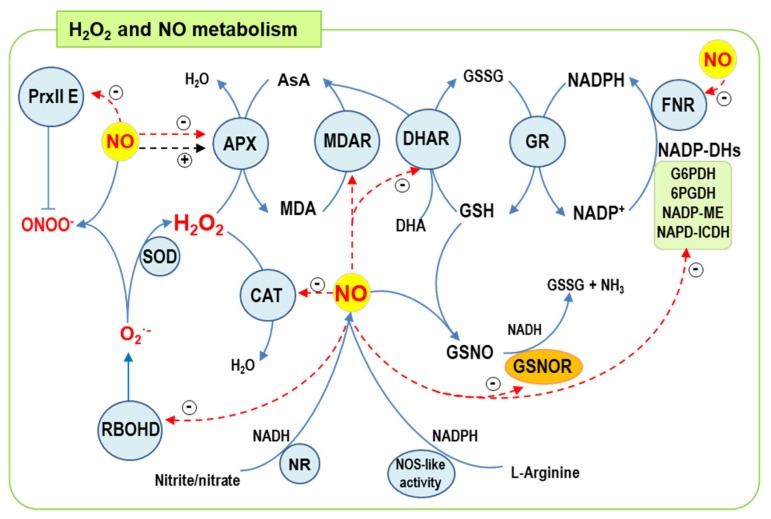
Model of the functional interaction between H_2_O_2_ and NO metabolism in plant cells. H_2_O_2_ is formed by either the one-electron reduction of O_2_^•−^ chemically or by SOD isozymes. H_2_O_2_ can be discomposed by different antioxidant systems depending of the subcellular compartments such as the peroxisomal catalase or a group of four enzymes which constitute the ascorbate glutathione cycle present in chloroplasts, cytosol, peroxisomes and mitochondria. NO can be generated either by NADPH and l-arginine-dependent NO synthase (NOS)-like activity or by nitrate reductase (NR). Dashed arrows indicate the NO effect on enzymatic activities; red: inhibition, black: enhancement.Blue line with a straight line at the end indicates peroxynitrate reduction by PRX II E. Abbreviations: APX—ascorbate peroxidase. AsA—ascorbatic acid. CAT—catalase. NO—nitric oxide. ONOO^−^—peroxynitrite. PRX II E—peroxiredoxin II type E. 6PGDH—6-phosphogluconate dehydrogenase. DHA—dehydroascorbate. DHAR—DHA reductase. GSH—reduced glutathione. FNR—ferredoxin-NADP reductase. NADP-DHs—nicotinamide adenine dinucleotide phosphate dehgyrogenases. G6PDH—glucose-6-phosphate dehydrogenase. GR—glutathione reductase. GSNO—S-nitrosoglutathione. GSNOR—GSNO reductase. GSSG—oxidized glutathione. H_2_O—water. H_2_O_2_—hydrogen peroxide. MDA—monodehydroascorbate. MDAR—monodehydroascorbate reductase. NADH—nicotinamide adenine dinucleotide. NADPH/NADP^+^—nicotinamide adenine dinucleotide phosphate. NADP-ICDH—NADP-isocitrate dehydrogenase. NADP-ME—NADP malic enzyme. NH_3_, ammonia. NR—nitrate reductase. O_2_^•−^—superoxide anion. RBOHD—respiratory burst oxidase homolog type D. SOD—superoxide dismutase.

The ASC-GSH cycle is comprised of four enzymes: ascorbate peroxidase (APX), dehydroascorbate reductase (DHAR), monodehydroascorbate reductase (MDAR), and glutathione reductase (GR). These enzymes require the presence of non-enzymatic antioxidants ascorbate (ASC) and glutathione (GSH) (Figure 4), which scavenge virtually all types of ROS, while the chloroplast contains the main cellular ascorbate pool in the micromolar range [35]. In this cycle, NADPH acts as an electron donor provided by the fumarate and nitrate reduction (FNR) protein during photosynthesis. NADPH is also supplied by a group of NADP-dehydrogenases (NADP-DHs), including NADP-isocitrate dehydrogenase (NADP-ICDH), NADP-malic enzyme (NADP-ME), glucose-6-phosphate dehydrogenase (G6PDH), and 6-phosphogluconate dehydrogenase (6PGDH), with the latter two belonging to the oxidative pentose phosphate pathway. These NADP-DHs function under dark phase and stress conditions.

Prx and Trx systems are involved in a complex redox homeostasis network aimed at protecting the chloroplast against oxidative damage [36,37]. Prxs are thiol-based peroxidase enzymes, such as H_2_O_2_, alkyl hydroperoxide (ROOH), and ONOO^−^, present on various peroxide substrates. With a catalytic efficiency in the range of 10^5^–10^8^ M^−1^ s^−1^ greatly depending on thiol regeneration, Prxs contain one or two essential cysteines in conserved sequences and can be classified into four main groups in higher plants: 1-Cys Prxs, containing one conserved cysteine; 2-Cys Prxs; Prxs Q; and Prxs type II, containing two catalytic cysteines [38]. Arabidopsis has been observed to contain Prxs and cysteines (2-CysPrxA, 2-CysPrxB, PrxQ, and PrxIIE) located in chloroplasts [39]. 

Trxs which are small thiol-disulphide proteins with 14 kDa, catalyze reversible disulphide bond formation, which facilitates the functional regulation of many proteins. Arabidopsis encloses twenty Trx isoforms, six of which (Trxs f1-2, m1-4, x, y1-2, z, and h) are present in chloroplasts [40]. Chloroplastic Trxs, which use a ferredoxin or NADPH reductant system, are designed as ferredoxin (Fd)-dependent Trx reductase (FTR) or NADPH-dependent Trx reductase (NTR) [37]. Both types of Trx regulate chloroplast redox homeostasis and interact with Prxs to establish a redox signal cascade and to modulate thiol groups [36]. A Trx combined with nitroreductase (NTR)at the C terminus is called NTRC, which, when involved in redox regulation, appears to affect starch and chlorophyll biosynthesis [41]. 

^1^O_2_ scavenging chiefly occurs through reactions with biomolecules such as tocopherols [42], carotenoids [43], and membrane lipids [44]. β-carotenes are involved in ^1^O_2_-mediated chloroplast retrograde signaling [45]. ^1^O_2_ and O_2_^•−^ have a short life-time and perform signaling functions due to their involvement in other signaling components such as proteins EXECUTER 1 and 2 [46], which points to the involvement of ^1^O_2_ in nuclear gene modulation. The ^1^O_2_-producing Arabidopsis *flu* mutant is totally defenseless against paraquat stress and enhanced chloroplast APX expression, suggesting an interaction between ^1^O_2_ and H_2_O_2_ signals [47]. Xanthophylls, such as violaxanthin, zeaxanthin and antheraxanthin, components of the xanthophyll cycle, provide protection against excessive ROS production [48]. Vitamin E (α-tocopherol) scavenges ^1^O_2_ [49] to form α-tocopheryl quinine [50], while tocopherols play a major role in inhibiting lipid peroxidation in thylakoid membranes by quenching lipid radicals [51]. 

#### 2.1.3. Stromules and ROS

Stromules are tubular plastid extensions [52] whose synthesis is rapidly induced by ROS, sugar, hormones and pathogen effector proteins. The formation of stromules is directly dependent on photosynthetic reactions and chloroplast redox status [53]. They deliver ROS directly to the nucleus and signaling activity through chloroplast metabolite sequestration which regulates the number of nuclei and plastids in cells [54]. However, H_2_O_2_ synthesized in the chloroplast in response to light reactions is transferred to nuclei without stromule formation when the chloroplast is adjacent to the nucleus [54]. An in situ increase in H_2_O_2_ synthesis when the chloroplast near the nucleus is exposed to 405 nm laser light has been found to lead to a transfer of H_2_O_2_ to the nucleus [55]. Furthermore, the generation of chloroplastic ROS indirectly affects nuclear gene expression due to oxidative post-translation modifications (PTMs) of the 3´-phosphoadenosine 5´-phosphate (PAP) phosphatase SAL1 enzyme, which is inhibited by H_2_O_2_-dependent oxidative PTMs. Inactivated PAP acts as a secondary messenger and assists in the transfer of information from the chloroplast to the nucleus [56].

### 2.2. Mitochondrial ROS/NO Generation and Scavenging System

Mitochondria contain a respiratory electron transport chain (ETC) comprised of four key complexes: NADH dehydrogenase (complex I), succinate dehydrogenase (complex II), cytochrome c reductase (complex III), and cytochrome c oxidase (complex IV), in addition to two mobile electron transporters, cytochrome c, and ubiquinone [57]. The respiratory ETC generates an electrochemical potential which leads to ATP synthesis by ATP synthase. As with the chloroplast, the electron is transferred from complexes I and III to O_2_ under abiotic stress conditions with a concomitant production of O_2_^•−^, which is dismutated to H_2_O_2_ by MnSOD and quenched by APXs or Prxs. Unlike chloroplasts, mitochondria have only one Prx called Prx II F, while mitochondria have two Trxs (Trxo1 and Trxo2) which can activate alternative oxidase (AOX) [58]. To function effectively, Trxs require NADPH which is supplied by the flavoenzyme NTR. Likewise, H_2_O_2_ reacts with reduced Fe^2+^ and Cu^+^ to produce highly toxic ^•^OH through a Fenton reaction. It is worth noting that the alternative oxidase (AOX) enzyme provides another route to the cytochrome pathway which facilitates its transfer to O_2_ to form H_2_O and dissipates energy in the form of heat to prevent the formation of ROS [59] especially under stress conditions [60]. Approximately 2–4% of O_2_ absorbed by the mitochondrial membrane is reduced to O_2_^•−^. 

Under stress conditions, the active form of the AOX homodimer can be reduced to the oxidized inactive form of the AOX monomer, which disrupts the mitochondrial ETC, resulting in enhanced gene-encoding AOX expression and other target such as mitochondrial dysfunctional stimulon (MDS) gene. Enhancement of AOX activity directs free electrons to O_2_, reduces ROS production and counters changes in redox homeostasis. This reaction mixture is transduced to two endoplasmic reticulum (ER) transcription factors (TFs), Arabidopsis NAC domain-containing protein 13 (ANACO13) and ANACO17. These TFs are then translocalized to the nucleus in response to stress signals [61]. However, mitochondrial DNA can be damaged by ROS over-production which eventually alters repair and replication processes [62]. Meanwhile, carbon monoxoide and cyanide can also cause complex IV to be suppressed, leading to an increase in ROS production [63]. In maize plants, an increased mitochondrial ROS generation under water deficit conditions facilitates rapid acclimation due to positive interactions between ROS and abscisic acid [64]. The integration of ROS and phytohormones has been reported to mediate mitochondrial H_2_O_2_ production under heat and oxidative stress conditions [65]. In plants, the final stage of ascorbate synthesis is catalyzed by the inner membrane-bound enzyme l-galactono-1,4-lactone dehydrogenase (GalLDH) which oxidizes l-galactono-lactone to ascorbic acid [66] and supplies an electron to complex IV (terminal oxidase) in the mitochondrial matrix [67]. 

Mitochondria generate NO, which is produced by chemical reduction from nitrate and not by enzymatic activity [68]. NO counters mitochondrial ROS overproduction [62], while its production is currently thought to involve complexes III and IV, while its removal by AOX can occur under normoxia conditions [69]. It also impacts the functioning of certain mitochondrial enzymes, as enrichment of pepper fruits with NO has been found to increase GalLDH gene expression and enzyme activity, with a concomitant increase of 40% in ascorbate content [70]. 

Mitochondria also generate extensions called matrixules which appear to play an, as of yet, little understood role in the ROS signaling mechanism [1]. 

### 2.3. Peroxisomes

Plant peroxisomes are single membrane-bound organelles characterized by an adaptable and active nitro-oxidative metabolism [15,71,72,73]. ROS/RNS are produced in many peroxisomal metabolic pathways such as β-oxidation, photorespiration, purine, and polyamine metabolism, as well as auxin and jasmonic acid biosynthesis. Table 2 summarizes how peroxisome organelles, with their complex battery of enzymes, are involved in a diverse range of biochemical pathways which are shared with other subcellular compartments. To regulate ROS production, peroxisomes contain a set of antioxidant systems including catalase, SOD isozymes, four ASC-GSH cycle components, and a Prx-like protein. Peroxisomes are produced in a matrix, containing catalase, SOD, GR, DHAR, and Prx-like proteins, or a membrane-bound APX, with the exception of MDAR, which is present in both these peroxisomal locations. To support the ASC-GSH cycle, peroxisomes contain GSH, ascorbate, and several NADPH-generating enzymes including NADP-ICDH, G6PDH, and 6PGDH [71,74,75]. 

NO was first identified in plant peroxisome organelles, in which a NOS like activity oxidizes L-arginine to NO using NADPH as a donor requiring Ca^2+^ and calmodulin. Peroxisomal NO is necessary for various physiological functions such as pollen tube growth, leaf senescence, auxin-induced root organogenesis, and abiotic stress responses such as salinity, cadmium, and lead. Peroxisomes contain ONOO^−^ and GSNO, while peroxisomal proteins, such as catalase, MDAR, GOX, hydroxypyruvate reductase, and malate dehydrogenase, prone to tyrosine nitration and *S*-nitrosation, have also been identified. Like chloroplasts and mitochondria, peroxisomes appear to generate peroxules under ROS stress conditions [76]. 

### 2.4. Vacuoles 

Vacuoles, which contain abundant antioxidants and play a multifunctional role in plant metabolism, are among the largest redox homeostasis maintenance sites in response to elevated levels of ROS under abiotic stress conditions [77]. Vacuolar sequestration of ROS is supported by class III peroxidases localized at the inner tonoplast membrane. Peroxidase attacks H_2_O_2_ generating ^•^OH via the hydroxylic cycle, while proteomic analysis has revealed that RBOH is localized in the tonoplast region [78]. Cellular H_2_O_2_ is diffused through the tonoplast either directly or via aquaporins in vacuoles [79], which are mainly composed of flavonoids, ascorbate, and peroxidases present on the inner surface of the tonoplast [80]. Enhanced vacuolar ascorbate content is involved in ROS detoxification under high light [81] and drought stress [82] conditions. Despite its low content, GSH is accumulated in vacuoles in oxidized form (GSSG) in response to oxidative stress [83]. Soluble sugars (sucrose) and sugar alcohols (fructans) are sequestered in vacuoles in response to oxidative stress [77]. Fructans protect membranes against freezing and drought stress by scavenging ^•^OH, a reaction which is triggered by enhanced phenolics, GSH and ascorbate present in vacuoles, suggesting that phenolics and fructans are synergistically active [84]. Finally, a recent study found that ROS overproduction causes photodamage to chloroplasts, which can induce chlorophagy, leading to vacuolar digestion of entirely photodamaged chloroplasts [85]. 

### 2.5. ROS and NO in Cell Membranes 

The plasma membrane is an important center of ROS production due to the presence of membrane-bound NADPH oxidase, also known as the respiratory burst oxidase homolog (RBOH). RBOH can transfer free electrons from its intracellular region to molecular oxygen via the apoplast through the plasma membrane [86]. The complex RBOH protein is comprised of a membrane-bound RBOH (molecular weight of 105 to 112 kDa) and its cytosolic regulator Rop (Rho-like protein); as well as an integral plasma membrane protein consisting of six transmembrane domains (TMD1-6) connected by five loops (A to E). The TMD-3 and TMD-5 domains contain a pair of His residues required to bind to the heme group, FAD and NADPH hydrophilic domains, and two N-terminal Ca^2+^-binding EF-hand motifs, indicating that RBOH activity is regulated by Ca^2+^ [87,88]. In addition to this complex structure, regulatory elements, including Ca^2+^, phosphorylation, phosphatidic acid, and NO, are also involved such as Ca^2+^-dependent andCa^2+^-CaM-dependent protein kinases, as well as a *S*-nitrosation mechanism [87]. 

Although their number varies according to the plant species, RBOH genes belong to a multigene family composed often members in *Arabidopsis thaliana*, nine in rice (*Oryza sativa*), eight in tomato (*Solanum lycopersicum*) and sweet orange (*Citrus sinensis*) and seven in common grapevine (*Vitis vinifera*). RBOH activity is involved in many plant processes including cell growth, plant development, stomatal closure, pollen tube growth, abiotic stress, symbiotic interactions, and pathogen responses [89]. It is also involved in fruit ripening [90] and stone cell lignification in fruit-producing trees [91]. RBOH’s functional diversity suggests that each isozyme is characterized by a particular specialization. 

#### 2.5.1. Apoplasts

The apoplast, linking the environment and cells, is the plant cell region outside the plasma membrane. Apoplastic spaces are more complex than one would expect due to the presence of various components involved in ROS metabolism. These components, which have been found in the apoplast of different plant species, include APX, GR, guaiacol-dependent peroxidase, SOD isozymes, GSH, diamine oxidase (DAO), and polyamine oxidase [92], in addition to NO [93] which can undergo *S*-nitrosation [94]. 

In many cases, apoplastic ROS metabolism is crucial for processes, such as axillary bud outgrowth [95], signal and nutrient transfer [96], abiotic stress defenses [97], and microbial pathogens, in which the RBOH plasma membrane is involved [89]. Under water deficit and salinity stress conditions, apoplastic ROS are enhanced, leading to cell wall strengthening and elongation, as well as cell growth [98]. Apoplastic ROS production is also affected by chloroplastic ROS generation which, in turn, modulates nuclear gene expression [99], a fine example of retrograde signaling.An increase in NO has also been observed to boost Fe immobilization in Fe-deficient root apoplasts by decreasing cell wall pectin methylation [100]. 

ROS signaling in apoplastic spaces involves local signals including: (i) sensing mechanisms directly via oxidative PTMs and (ii) sensing mechanisms indirectly via oxidation of metabolites present in apoplastic regions [101]. Apoplastic spaces also contain a few cysteine-rich peptides (CRPs) namely cysteine-rich receptor-like kinases (CRKs) [102]. These findings that corroborate the role of CRKs in ROS sensing show that: (i) membrane proteins contain conserved cysteines; (ii) apoplastic ROS accumulation controls transcriptional regulation [96]; and (iii) that they also play a role in *crk* mutant plants [102]. A set of CRKs, which has been reported to interact with RBOHs, are also involved in ROS sensing and signaling [103]. Another cysteine-rich protein GRIM REAPER (GRI) and a receptor-like kinase (RLK) POLLEN RECEPTOR LIKE KINASE 5 (PRK5) have been reported to combine to form an important ROS signaling unit [96,104]. GRI proteins are cleaved by the metacaspase 9 enzyme, resulting in the formation of a 12-amino acid GRI peptide which binds with its receptor PRK5 and initiates ROS-dependent programmed cell death [96]. 

Trxs, which are involved in regulating redox status under various environmental cues via thiol-disulfide interchange reactions, are also present in the apoplast [105]. Secretion of polyamines in the apoplast under abiotic stress conditions modulates its redox status [106], while apoplastic regions contain ROS-producing enzymes such as apoplastic peroxidases and polyamine oxidases (PAOs) [107]. Two peroxidases, PRX33 and PRX34, which play a key role in apoplastic ROS generation under biotic stress conditions, have been identified in the apoplastic region of Arabidopsis plants [108]. PAOs stimulate spermine and spermidine catabolism, while H_2_O_2_ is simultaneously produced. The H_2_O_2_ produced plays a pivotal role in plant biotic and abiotic stress [109] and development [110].

#### 2.5.2. Cell Walls

Cell walls are another major location for the accumulation of ROS [111], whose production is boosted by enzymes such as class III peroxidase, amine oxidase, quinone reductase and oxalate oxidase [112]. For example, with their dual hydroxylic and peroxidative cycles, class III peroxidases can either generate ROS or oxidize cell wall aromatic compounds in proteins and phenolics which are free or linked to polysaccharides [113]. Cell wall peroxidase can oxidize NADH and stimulate O_2_^•−^ production, thus activating adaptive plant responses to abiotic stress conditions, particularly hyperthermia and drought [114]. Diamine oxidase is involved in cell wall ROS generation, while diamines, including spermine, spermidine, cadaverine, and putrescine, are used to reduce quinine to peroxide through autoxidation [111]. ^•^OH production in vivo and in vitro has been observed in various plant cell walls [115,116], while O_2_^•−^ and H_2_O_2_ generation in maize root cell walls has been confirmed by histochemical analysis. O_2_^•−^and ^•^OH react with the methyl group of polysaturated fatty acids to form conjugated dienes, hydroperoxides, and peroxy radicals, which, in turn, react with metal ions such as Fe^2+^, while the lipid radicals formed initiate a further chain reaction [117]. 

NO also affects cell wall metabolism [118] and induces Cd tolerance by increasing pectin and hemicellulose content in the root cell wall [119]. In the internal cell wall of rice, crosstalk between NO and ethylene has been found as well as phosphorus (P) remobilization, especially under P-deficient conditions [120]. In addition, *S*-nitrosoglutathione (GSNO) promotes cell wall remodeling and induces root hair formation in Arabidopsis [121]. 

Table 3 shows some representative examples of ROS/RNS metabolism in the different subcellular compartments mainly chloroplasts, mitochondria, peroxisomes, and cell wall under different stressful conditions including salinity.

## 3. ROS and Signaling Molecules

Biological responses to ROS depend on the type of ROS, signal magnitude, formation site and crosstalk with other signaling molecules such as MAPK, NO, and Ca^2+^. 

### 3.1. Redox Modulation of MAPKs

The MAPK cascade is conserved in eukaryotes which initiate the signaling process when MAPK kinase kinase (MAPKKK) activates MAPK kinase (MAPKK) which, in turn, activates MAPKs. The relationship between the MAPK cascade and ROS has been described in various studies; exogenous H_2_O_2_ mediates the activation of several components in the MAPK cascade by, for example, inactivating MAPK repressors, although, ROS have been observed to respond when this cascade is manipulated [135]. The cascade is involved in many physiological processes such as cell division, plant growth and development, stomatal movement, abiotic stress responses, and pathogen defenses.

MAPK cascades are considerably involved in retrograde signaling from chloroplast to the nucleus of the stressed plant cells [136]. This network is a fundamental signaling component in the conversion of ROS signals to protein phosphorylation. Adachi et al. [137] identified a W-box in the promoter region of the tobacco *RBOH* gene, as well as WRKY transcription factors, both of which, they suggest, are phosphorylated by MAPK, thus revealing further crosstalk between MAPK phosphorylation and RBOH. 

### 3.2. Redox Stimulation of Ca^2+^Mobilization

Cellular Ca^2+^ homeostasis is strictly regulated given the importance of Ca^2+^ as a second messenger which remains local or is transmitted throughout the whole cell in milliseconds to seconds. This regulation involves Ca^2+^ pumps, channels, cation exchangers, and Ca^2+^-binding proteins, as the Ca^2+^ cation regulates multiple cellular activities. Moreover, crosstalk between Ca^2+^ and other signaling molecules, including ROS and NO, which affects gene expression and Ca^2+^-dependent protein kinases, takes place under biotic and abiotic stress conditions [138]. 

Mutual crosstalk between ROS and Ca^2+^ signaling pathways can be both antagonistic and synergistic, depending on the ROS species, target protein type, duration, dosage, and tissue type. The formation and removal of ROS and their interaction with Ca^2+^ signaling are complex mechanisms which provide an excellent model to explore the molecular players involved in the interplay between ROS and other signaling cascades. An enhanced metabolic rate can lead to higher levels of O_2_, which increases the leakage of respiratory ETC and ROS concentrations. ROS generation in mitochondria is directly associated with the metabolic rate, and Ca^2+^ accumulation reduces the level of ROS generated by complexes I and III under normal conditions, while increasing ROS formation when these complexes are suppressed by pharmacological agents. Ca^2+^ may stimulate three dimensional (3-D) conformational alterations in respiratory ETC complexes, which further induces ROS production in mitochondria [139]. When membrane potential is enormous in the absence of ATP synthesis, Ca^2+^ uptake reduces ROS formation. When membrane potential is in a depolarized range in the presence of ATP synthesis, ROS production is triggered which may be dependent on Ca^2+^ load. With a high Ca^2+^load in mitochondria, ROS generation independently enhances the mitochondrial metabolic rate [140]. The overall effect of Ca^2+^ on ROS production and eradication depends on subcellular compartments and cell tissue. CaM, a Ca^2+^binding protein with approximately 16 kDa, modulates antioxidative enzyme activity and is associated with ROS homeostasis. The suppressed or activated status of redox regulation may alter the activity, transcript level, open channel and trafficking [141]. Receptor-triggered Ca^2+^ signals are important for fully functioning cells involved in the discharge of Ca^2+^ from its storage site and the entry of Ca^2+^ through plasma membrane channels. The two main categories of channel proteins associated with receptor-triggered Ca^2+^ entry signals are transient receptor potential and store-operated Ca^2+^ channels [142]. 

With regard to free-radical NO generation, Ca^2+^ needs to be present in crosstalk with H_2_O_2_ for development purposes and in response to a diverse range of abiotic stresses [143]. At the subcellular level, peroxisomes are a good example of NO production, for whichCa^2+^ together with CaM and NADPH redox power are strictly required [75].

## 4. Redox-Sensitive Genes

Redox balance in stressed cells is maintained by the ASC-GSH cycle which plays important role in regulating H_2_O_2_ concentrations. Oxidant-dependent sulfhydryl GSH:GSSG cysteine conversion plays a key role in stress management by regulating redox homeostasis and also controls the function of various transcription factors [144]. In addition, redox-sensitive gene expression needs to be regulated by ROS for detoxification purposes. Oxidative stress stimulates cell signaling [145], while H_2_O_2_ signaling and receptor-like kinase activation play a significant role in regulating redox-sensitive gene expression [146]. H_2_O_2_ signaling and accumulation also play an important role in activating enzymatic antioxidant-encoding gene expression and can be effectively used to regulate stress-responsive gene activity to withstand harsh environmental conditions. Furthermore, O_2_^•−^ has specific targets including *WRKY46*, *WRKY28*, and *WRKY22*. Redox-sensitive genes include glutathione peroxidase (*GPX*), catalase (*CAT*), *SOD2*, quinone reductase, heme oxygenase-1, ferritin, *GR*, thioredoxin reductase, thioredoxin, metallothionin, cycloxygenase-2, and γ-glutamylcysteine synthase which is required for GSH synthesis [147]. 

### 4.1. Role of ROS and NO in the Regulation of Transcription Factors (TFs)

In eukaryotic cells, TFs are regulated by complex redox-sensitive systems, consisting of many systematically ordered elements, including GTPase, phosphatase, and lipase, as well as phospholipid-dependent and MAP kinases. ROS-regulated TFs contain reactive cysteine thiol groups whose redox status is affected such as Ca^2+^-ATPase, collagenase, and SRC tyrosine kinases. However, ROS not only regulate pre-existing protein activity but also induce the expression of certain genes that control the gene expression patterns of other signal transduction networks. Interest in how ROS and NO regulate gene expression under stress conditions and in identifying the TFs involved has been increasing. Available information indicates that highly complex signaling networks involvingH_2_O_2_ and NO, as well as phytohormones such as ABA, JA, and SA, are implicated in signaling cascades. Recently, up to sixty H_2_O_2_-responsive TFs involved in stresses such as cold, drought, heat, high light, and pathogens, were identified [148]. On the other hand, GSH enhances tolerance to Cd stress in tomato by increasing the antioxidant system, NO and *S*-nitrosothiols via a redox-dependent upregulation of TFs such as the ethylene-responsive TFs ERF1, ERF2 and myeloblast 1 (MYB1), as well as other stress response genes [149]. In the case of *Arabidopsis thaliana* exposed to GSNO, RNAseq analysis reveals overexpression of TF groups including five members of the WRKY gene family involved in pathogen and abiotic stress responses, four members of the MYBTF family, heat stress TFs, and the transcription factor-binding TF GT-1 which binds to the cis-acting element BoxII [32]. Recently, Hartman et al. [150] identified a mechanism of response to hypoxia in Arabidopsis in which ethylene boosts ERF VII TF stability by increasing NO-scavenger phytoglobin 1 in order to facilitate plant survival.

### 4.2. Regulation of Iron-Responsive Element-Iron Regulatory Protein (IRE-IRP) System 

In eukaryotic cells, the IRE-IRP system consists of iron responsive elements (IREs) and iron regulatory proteins (IRPs). Iron (Fe) is an important nutrient which plays a significant role in cellular metabolism and morphogenesis. It acts as a co-factor in major enzymes such as ribonucleotide reductase, cytochrome P450, and leghemoglobin, as well as those involved in ROS metabolism including cytochrome c oxidase (COX), xanthine oxido-reductase, catalase, APX, Fe-SOD, and peroxidase [151]. However, through the Fenton reaction, the presence of higher concentrations of Fe leads to ROS synthesis. Nevertheless, cellular Fe homeostasis, which regulates gene expression at the post-transcriptional level, is critical for Fe transportation and accumulation inside the cell. 

IRP1 and IRP2, which interact with IRE mRNA in the 3ʹ and 5ʹ untranslated regions (UTRs), are necessary for Fe homeostasis. Transferrin receptor-1 (TFR1) regulates Fe movements in cells and ferritin, as well as cellular Fe storage [152]. The fate of mRNA is decided by binding IRP1 and IRP2 to IRE which has a stem-loop structure consisting of roughly 30 nucleotides with a mandatory core5ʹ-CAGUG-3ʹ sequence for IRP binding. IRP binding to IRE TFR1 in the untranslated region enhances mRNA stability and the protein expression, while IRP binding to ferritin blocks mRNA translation and decreases protein expression. 

It is crucial to monitor soil mineral absorption by root cells for plant growth and development purposes [153]. Any defect in iron-regulated transporter 1 (IRT1), a major regulator of plant iron homeostasis, can cause severe chlorosis [154]. The *IRT1* gene is highly expressed in iron-deficient root cells, resulting in elevated levels of iron absorption which improves growth and development under iron-starved conditions. IRTI is involved in the absorption of other minerals, including Zn, Mn, Ca, Co, and Ni [155], and in the main toxic metal entry pathway in iron-deficient plant cells. Various transcriptional factors bind directly to the IRT1 promoter in root epidermal cells and are induced by low concentrations of iron [156]. 

Plant root secretion of Fe-mobilizing phenolic compounds facilitates Fe detoxification phenomena [157]. Fe^3+^ ions are reduced to Fe^2+^ ions by ferric chelate reductase enzyme FRO2 and then transported through the rhizodermal cell membrane by IRT1 [158]. On the other hand, iron deficiency activates transcriptional changes, which stimulate Fe uptake, leading to over-accumulation of Fe and other divalent metals, namely Zn, Cd, and Mn, due to the low specificity of IRT1, whose recycling is used as a posttranslational technique by plants [155]. In *Arabidopsis* plants, IRT1 is sequestered and gradually degraded by ubiquitination and phosphorylation, a process triggered by IRT1 binding to metal ions other than Fe [159]. IRT1 proteins have been observed in the initial stages of early endosome/trans-Golgi network (EE/TGN) compartmentalization in response to ubiquitin- and clathrin-dependent late endosomes [155]. Despite regulation by RING-typeE3 ligase IDF1 [160], IRT1 ubiquitination is unaffected by primary substrate iron availability [155]. Nitrate root uptake increases pH in the rice rhizosphere, which triggers Fe deficiency. This is due to an increase in H_2_O_2_ induced by RBOH1, with a concomitant boost in lignin biosynthesis and low phenolic secretion, which blocks apoplastic Fe mobilization efficiency [161]. 

NO also preserves Fe homeostasis through crosstalk with ethylene and auxin phytohormones, ferritin and frataxin and through its ability to form nitrosyl iron complexes [162]. New elements, such as GSH, GSNO, and GSNO reductase, have been found in the complex relationships with the Long distance iron signal which moves from shoots to roots through the phloem to regulate Fe homeostasis [163]. GSNOR activity, in particular, has been found necessary for Fe homeostasis, especially under Fe-toxicity conditions in roots [164]. 

## 5. ROS and NO Intermediates in Signal Transduction

As outlined above, ROS and NO play a crucial role in signaling mechanisms, which modulate plant growth and development, transpiration, hormonal regulation, germination, stomatal gaseous exchange, flowering, fruit ripening, defense responses, and programmed cell death (PCD) [41].

Although the endogenous sources of enzymatic NO in higher plants remain a subject of debate such as it was mentioned previously, its presence and function in plant cells are clear [17]. NO performs a signaling function mainly through the PTMs, *S*-nitrosation, nitration, and metal nitrosylation, in order to regulate gene expression and its interaction with phytohormones [165].

### 5.1. Regulatory Role of NO PTMs, S-Nitrosation and Nitration in ROS Metabolism

In higher plants, *S*-nitrosation and nitration are the most studied NO PTMs [166,167]. While S-nitrosation involves reversible covalent binding of a NO group to the thiol (–SH) side chain of a susceptible Cys, nitration adds a nitro group (–NO_2_) to susceptible amino acids, with tyrosine (Tyr) being the most studied. In both cases, the PTM positively or negatively affects protein function but could also have no functional impact. 

Over the last ten years, significant advances have been made in the regulation by NO of ROS metabolism in plants. Figure 4 shows a simple model of the most important ROS metabolism enzymes modulated by NO PTMs. Catalase, exclusively present in peroxisomes, is negatively regulated by both nitration and *S*-nitrosation [168]. On the other hand, *S*-nitrosation, which inhibits O_2_^•−^-generating RBOHD in Arabidopsis [169], does not affect SOD isozymes. However, nitration triggers the inhibition of chloroplast Fe-SOD3 (FSD3), mitochondrial Mn-SOD1 (MSD1), and peroxisomal Cu,Zn-SOD (CSD3) [170]. Different components of the ASC-GSH cycle present in subcellular cytosol, chloroplast, mitochondrial and peroxisomal compartments which regulate H_2_O_2,_ are differentially affected by nitration and *S*-nitrosation. Nitration inhibits, while *S*-nitrosation triggers an increase in APX activity [167]. Although both nitration and *S*-nitrosation inhibit MDAR activity, GR function remains unaffected by either PTM [167]. As mentioned above, Prxs are a complex family of antioxidant enzymes involved in thiol peroxidase activity on multiple peroxide substrates including H_2_O_2_, alkyl hydroperoxide (ROOH), and peroxynitrite (ONOO^−^), which are present in almost all cellular compartments [171]. In Arabidopsis, chloroplastic peroxiredoxin II E is involved in peroxynitrite reductase activity which is inhibited by *S*-nitrosation [172]. NADPH-generating enzymes maintain redox state and support antioxidant enzymes, such as GR, as well as O_2_^•−^ and NO generation by RBOH and NOS-like activity, respectively. These NADP-DHs are differentially modulated by nitration/*S*-nitrosation: chloroplast FNR is inhibited by nitration, NADP-ICDH and NADP-ME are inhibited by both nitration and *S*-nitrosation [173], while G6PDH and 6PGDH appear unaffected [174]. Remarkably, the key enzyme *S*-nitrosoglutathione reductase (GSNOR), involved in physiological and stress processes [175], which regulates GSNO content and consequently *S*-nitrosation cellular capacity, is inhibited by *S*-nitrosation [176].

### 5.2. NO-Responsive Genes

Although NO regulates the expression of specific genes [177], high-throughput sequencing methods, called short-read massively parallel sequencing or RNA-seq, have advanced our knowledge of NO as a key regulator of gene expression reprogramming. Using NO donors, such as GSNO and NO gas, as well as Arabidopsis plants exposed to GSNO through the root system, 1945 responsive genes differentially expressed in leaves and roots,114 of which corresponded to only one of these organs, were identified [178]. Modulation during pepper fruit ripening of roughly 2987 genes, 498 of which were modulated by NO, has also been studied [41]. 

### 5.3. NO and Crosstalk with Other Plant Growth Regulators

NO, produced in plants to combat pathogen attacks, is directly involved in defense-related signaling pathways via secondary messenger protein kinases, cyclic AMP, signaling lipids, Ca^2+^, cyclic GMP, and various plant hormones [179]. It also contributes to processes such as hypersensitive response reactions and systemic resistance [180]. 

Functional molecular analysis of NO-responsive genes in defense pathways has been used to identify encoding proteins for secondary metabolism and pathogen-related proteins [181]. Skelly and Loake [86] have fully characterized proteins involved in *S*-nitrosation and tyrosine nitration, with non-expressor of pathogenesis-related gene 1 (*NPR1*) and AtRBOHD providing new insights into the role of NO in plant defenses. Many distinctive functions have been studied with regard to ROS-NO interactions, leading to the generation of related molecules such as NOx, ONOO^−^, N_2_O_3_, and NO_2_. They also act as oxidizing molecules on different proteins involved in NO-ROS signaling, resulting in structural and functional alterations in these proteins [182].

Yang et al. [183] have elucidated senescence-inducing ROS-NO crosstalk in litchi plants and have described the morphological, structural, PCD, and expression characteristics of senescence genes after treatment with ROS and NO. Their results show the distorted structure and morphology as well as the upregulation of *LcRBOH*, *LcMC-1*-like and *LcPirin* genes, which play a role in ROS- and NO-mediated senescence and leaf abscission in litchi plants [183]. NO and ROS have also been reported to play a vital role in signaling cascades and defense mechanisms in legumes and rhizobia [184]. Interaction between ROS and NO assisted by post-translational changes in nitrosated and sulfenylated proteins occurs during nitrogen-fixing symbiosis (NFS) [185]. In addition, glutamine synthetase 1, mainly nitrated innodules, is a critical enzyme involved in nitrogen fixation [186]; this post-translational initiation of glutamine synthetase 1 activity directly correlates NO signaling with N_2_ metabolism in nodules. In a study of inoculated *M. truncatula* plants treated with an RBOH oxidase activity inhibitor, the NO scavenger clearly showed overlapping signaling pathways mediated by these molecules [187]. Gene expression analysis has revealed that genes encoding for defense and secondary metabolites are up-regulated and altered during programmed cell death [188]. However, with the aid of transcriptomic analysis, *MtNR1*, *Mtns-Hb1,* and *MtNiR* genes have been observed to be upregulated in *M. truncatula* roots as a result of mediation by H_2_O_2_ of enzymatic transcriptional regulation to maintain NO homeostasis [184]. Similarly, ROS generation activates the NR pathway in *Arabidopsis* during plant pathogenesis, which leads to higher levels of NO production and affects RBOHD activity via *S*-nitrosation [169]. For instance, the connection between NO and ROS through overlapping signaling pathways results in negative NO feedback from ROS generation.

## 6. Conclusions

ROS/RNS, a fundamental feature of plant cell metabolism, increase nitro-oxidative damage when overproduced and prevent regulation by the corresponding antioxidant systems. Figure 1 shows a simple working model of ROS and RNS metabolism in plant cells under stress conditions. ROS/RNS generation is associated with virtually all cellular compartments, particularly chloroplasts, mitochondria, peroxisomes, and the plasma membrane, with inter-compartmental cooperation acting as a regulatory mechanism. Some compartments also interact with the nucleus through retrograde signaling which enables gene expression to be regulated. In the future, this relatively new area of research should provide new insights into the complex networks both inside and between cells, as well as a reappraisal of redox metabolism. Important new avenues of research are opening up in relation to ROS/RNS metabolism, particularly with respect to items such as hydrogen sulfide (H_2_S), serotonin and melatonin. Finally, the complex relationships involved in cellular ROS/RNS metabolism need to be looked at from a broad perspective and we particularly hope that this assessment provides non-specialist researchers and academics alike with a clear understanding of this field of research. 

## Figures and Tables

**Table 1:** Summary of main subcellular compartments and major pathways involved in ROS and RNS production in plant cells.

Site of Production	ROS/RNS Produced	Pathways Involved
Chloroplasts	O_2_^•−^, H_2_O_2_ ^•^OH, ^1^O_2_, ^•^NO, ONOO^−^	Light dependent photosynthetic reaction (PS I and PS II), Fenton reaction. SOD isozymes. NO synthase-like activity
Mitochondria	O_2_^•−^, H_2_O_2_, ^•^OH ^•^NO, ONOO^−^	Respiratory ETC (mitochondrial complexes I and III). Nitrate reduction
Peroxisomes	O_2_^•−^, H_2_O_2_, ^•^OH, ^•^NO, ONOO^−^	β-oxidation. Photorespiration. Sulfite detoxification pathway. Purine metabolism. Polyamine catabolism. NO synthase-like activity
Vacuoles	H_2_O_2_	ROS metabolism (enzymatic and non-enzymatic antioxidants).
Plasma membrane	O_2_^•−^, H_2_O_2_	RBOHs
Apoplast	H_2_O_2_ ^•^NO	ROS metabolism (SOD, GSH and NO interaction), thiol-disulphide network, cystiene rich kinases and polyamine catabolism (enhanced catabolism of spermine and spermidine). Nitrate reduction
Cell wall	O_2_^•−^, H_2_O_2_, ^•^OH, ^•^NO	Peroxidases, Oxidized NADH and diamino oxidases.

Abbreviations: O_2_^•−^—superoxide anion. H_2_O_2_—hydrogen peroxide. ^•^OH—hydroxyl ion. ^1^O_2_—singlet oxygen. ^•^NO—nitric oxide. ONOO^−^—peroxynitrite. PS I—photosystem I. PS II—photosystem II, SOD—superoxide dismutase.ETC—electron transport chain. ROS—reactive oxygen species. RBOH—respiratory burst oxidase homolog. GSH—reduced glutathione. NADH—nicotinamide adenine dinucleotide. The upper dot indicate in which atom is located the unpaired electron.

**Table 2:** The main enzymes involved in the generation of ROS and RNS in plant peroxisomes.

PeroxisomalEnzyme	ROS/RNS
Acyl-CoA oxidase	H_2_O_2_
Glycolate oxidase	H_2_O_2_
Urate oxidase (UO)	H_2_O_2_
Xanthine oxidoreductase (XOR)	O_2_^•−^
O_2_^•−^-generating membrane polypeptides	O_2_^•−^
Polyamine oxidase (PAO)	H_2_O_2_
Copper-containing amine oxidase (CuAO)	H_2_O_2_
Sulfite oxidase (SO)	H_2_O_2_
Sarcosine oxidase (SOX)	H_2_O_2_
L-arginine-dependent NO synthase (NOS) like activity	^•^NO

**Table 3:** Representative examples of ROS/RNS metabolism in the different subcellular compartments under different stressful conditions.

Sub-Cellular Compartment	Stress	Plant	ROS/RNS Metabolism	References
Plastids	Osmotic stress	*Arabidopsis thaliana*	Increase of ROS along with ABA biosynthesis and activation of retrograde signaling proteins *GUN1*, and *ABI4.*	Wilson et al. [122]
Chloroplast	Salt and osmotic stress	*Arabidopsis thaliana*	TOC apparatus (chloroplast membrane) regulated by the ubiquitin-proteasome system (E3ligase SUPPRESSOR OF PPI1 LOCUS1 (SP1)) to limit the import of photosynthetic apparatus components, and decline ROS accumulation and photo-oxidative damage, to maintain organelle proteome.	Ling and Jarvis [123]
Chloroplast	Salinity	*Nicotiana tabaccam*	Plastid-targeted cyanobacterial flavodoxin, a flavoprotein that prevents ROS accumulation in chloroplasts.	Lodeyro et al. [124]
Chloroplast	Salinity	*Arabidopsis thaliana*	Distorted grana and thylakoid structures and chloroplasticNADP-MDH along with cytosolic and mitochondrial NAD-MDH mediated export of NADPH and metabolites among cellular compartments to maintain redox homeostasis via malate valve situated atchloroplast envelope membrane.	Kandoi et al. [125]
Chloroplast	High light	*Arabidopsis thaliana*	Alternative oxidase led to enhance reductants in chloroplast stroma, respiratory O_2_ uptake, γ-aminobutyric acid (GABA) and N-rich amino acids while decline in photosystem II efficiency, intermediates of tricarboxylic acid (TCA) cycle and Calvin cyclesupported by dissipation of photosynthetic electron transport and primary metabolites.	Jiang et al. [126]
Chloroplasts Mitochondria, Peroxisomes	Drought	*Triticum aestivum*	Rise in the levels of oxidized modified proteins and induced oxidative damage was prevalent with the lower content of ascorbic acid in the chloroplast stroma.	Bartoli [67]
Mitochondria	Salinity	*Nicotiana tabaccam*	Disrupted shape from rounded to elongated, declined section area, branching of mitochondria, and emergence of triangular and rhomboid cristae, densification matrix, and contrasting membranes due to ROS effects.	Baranova et al. [127]
Mitochondria	High temperature	*Triticum aestivum*	Exogenously supplied NO (SNP, sodium nitroprusside) reduced oxidative stress markers such as malondialdehyde, H_2_O_2_ and O_2_^•−^	El-Beltagi et al. [128]
Mitochondria	Low temperature	*Capsicum annuum* L.	RNS and ROS mediated protein tyrosine nitration (NO_2_-Tyr) and lipid peroxidation, inducing nitrosative and oxidative stress.	Airaki et al. [129]
Mitochondria	Heat stress	*Laternula elliptica*	ROS induced loss of phosphorylation efficiency, enhanced mitochondrial oxygen demand and higher oxidative stress.	Heise [130]
Mitochondria and Peroxisomes	Salinity	*Cakile martima*	Accumulation of ROS markers malondialdehyde and CO- proteins) in mitochondria and peroxisomes along with enhanced antioxidant enzymes and ascorbate-glutathione cycle.	Amor et al. [131]
Peroxisomes	Heavy metal (Cr 200 µM)	*Zea mays* L.	H_2_O_2_-mediated glycolateoxidase and NADPH-regenerating machinery signified generation of oxidative stress, while restoration of adverse effects of ROS through exogenous NO application.	Kharbech et al. [132]
Peroxisomes	Heavy metal (Pb)	*Arabidopsis thaliana*	Overproduction of nitric oxide (NO), superoxide anion (O_2_^·−^) and peroxynitrite (ONOO^−^) in organelles thereby decreasing the catalase activity	Corpas and Barroso [74]
Cell wall, cytoplasm and organelles	Heavy metal (Cd)	*Arabidopsis thaliana*	Increase in soluble sulfide in cell compartments and ROS mediated oxidative damage of cell wall and organelles.	Guan et al. [133]
Cell wall, sub-cellular fractions and organelles	Fe deficiency stress	*Arachis hypogaea* L.	Exogenously applied NO (SNP) inhibited the malondialdehyde and ROS accumulation from different cell compartments.	Kong et al. [134]

Abbreviations: ABA—abscisic acid. Cd—cadmium. Cr—chromium. Fe—iron. GABA—γ-aminobutyric acid. H_2_O_2_—hydrogen peroxide. NAD-MDH—nicotinamide adenine dinucleotide-malate dehydrogenase. NADP—nicotinamide adenine dinucleotide phosphate. NADP-MDH—nicotinamide adenine dinucleotide phosphate-malate dehydrogenase. NO—nitric oxide. NO_2_-Tyr—Tyrosine nitration. O_2_—molecular oxygen. ONOO^−^—peroxynitrite. Pb—lead. ROS—reactive oxygen species. RNS—reactive nitrogen species. SP I—E3ligase SUPPRESSOR OF PPI1 LOCUS1.TOC apparatus—total organic carbon apparatus. TCA—tricarboxylic acid. SNP—sodium nitroprusside. O_2_^•−^—superoxide anion.
